# Factors affecting engagement between academic faculty and decision-makers: learnings and priorities for a school of public health

**DOI:** 10.1186/s12961-018-0342-9

**Published:** 2018-07-25

**Authors:** Nasreen S. Jessani, Sameer M. Siddiqi, Carly Babcock, Melissa Davey-Rothwell, Shirley Ho, David R. Holtgrave

**Affiliations:** Johns Hopkins Bloomberg School of Public Health, 624 North Broadway, Baltimore, MD 21205 United States of America

**Keywords:** Knowledge translation, Dissemination, Knowledge exchange, Evidence-informed decision-making, School of public health

## Abstract

**Background:**

Schools of public health (SPHs) are increasingly being recognised as important contributors of human, social and intellectual capital relevant to health policy and decision-making. Few studies within the implementation science literature have systematically examined knowledge exchange experiences within this specific organisational context. The purpose of this study was therefore to elicit whether documented facilitators and barriers to engaging with government decision-makers resonates within an academic SPH context. We sought to understand the variations in such experiences at four different levels of government decision-making. Furthermore, we sought to elicit intervention priorities as identified by faculty.

**Methods:**

Between May and December 2016, 211 (34%) of 627 eligible full-time faculty across one SPH in the United States of America participated in a survey on engagement with decision-makers at the city, state, federal and global government levels. Surveys were administered face-to-face or via Skype. Descriptive data as well as tests of association and logistic regression analyses were conducted using STATA.

**Results:**

Over three-quarters of respondents identified colleagues with ties to decision-makers, institutional affiliation and conducting policy-relevant research as the highest facilitators. Several identified time constraints, academic incentives and financial support as important contributors to engagement. Faculty characteristics, such as research areas of expertise, career track and faculty rank, were found to be statistically significantly associated with facilitators. The top three intervention priorities that emerged were (1) creating incentives for engagement, (2) providing funding for engagement and (3) inculcating an institutional culture around engagement.

**Conclusions:**

The data suggest that five principal categories of factors – individual characteristics, institutional environment, relational dynamics, research focus and funder policies – affect the willingness and ability of academic faculty to engage with government decision-makers. This study suggests that SPHs could enhance the relevance of their role in health policy decision-making by (1) periodically measuring engagement with decision-makers; (2) enhancing individual capacity in knowledge translation and communication, taking faculty characteristics into account; (3) institutionalising a culture that supports policies and practices for engagement in decision-making processes; and (4) creating a strategy to expand and nurture trusted, relevant networks and relationships with decision-makers.

## Background

### The use of research evidence in decision-making

A major focus of dissemination and implementation research is to enhance evidence-informed decision-making (EIDM) by exploring “*how, when, by whom, and under what circumstances evidence spreads*” to a variety of public health decision-making settings, including government agencies and legislatures [1]. Promoting EIDM therefore helps strengthen public health systems and institutions, assists in the attainment of domestic public health goals and contributes to global development agendas [2]. Research evidence produced by academic institutions, amongst others, has the potential to play a critical role in policy formulation, implementation, quality improvement, and policy maintenance and evaluation [3–7]. Similarly, practice-based insights and implementation strategies have important implications on research agenda-setting, measurement approaches and evaluation opportunities [8, 9].

In recognition of the value of EIDM, researcher engagement in decision-making has been growing [10]. In this context, academic researcher engagement refers to outreach and exchange between researchers and decision-makers in government on substantive issues related to researchers’ technical expertise or affiliation. Researchers benefit from this engagement as practice-based insights have the potential to enhance the responsiveness, reach and utility of research produced [11, 12] through knowledge translation (KT). However, there are a variety of factors that facilitate or hinder this kind of engagement.

### Current evidence on facilitators and barriers to engagement between researchers and decision-makers

Facilitators and barriers to KT in practice and policy sectors have been explored extensively within the implementation science literature from the perspectives of decision-makers [7, 13–16] as well as of researchers [14, 16]. Results indicate that barriers can be attributed to individual characteristics [17, 18], institutional environments [12, 13, 19, 20], and trusting relationships between both parties [13, 17, 21–23]. Common individual-level facilitators for researchers include knowledge of the policy-making process and stakeholder groups, access to decision-makers, perceived credibility [24], and training or mentorship in communication strategies [12, 13]. Common institutional-level facilitators include academic incentives for engagement, requirements or guidance from research funders, and alignment between research and legislative timelines [13, 25]. Barriers to engagement include non-receptive policy environments, practical limitations to implementation of policy options (i.e. resource constraints) and conflicts related to politically sensitive findings [13, 26]. Conversely, facilitators to engagement with decision-makers are often found to be related to research or evidence that was timely, relevant, had clear recommendations and was in line with the strategic goals of decision-makers [13, 21, 27].

However, a majority of the studies have focused on factors affecting research utilisation [7, 13, 20, 28–30] in contrast to knowledge exchange or engagement, as noted by Jacobson [20], who called for “*more investigation of the factors that promote or impede engagement in knowledge transfer*.” Furthermore, as noted in Orton’s systematic review [29], several stem from countries with universal healthcare systems such as United Kingdom, Canada and Australia. The context in the United States of America is notably quite different given the absence of such a health system and the influence of state-centred federalism on public health [31]. Furthermore, existing studies provide a comprehensive listing of individual (and at times institutional) facilitators and barriers to research utilisation but not the association between these and their combined effects on engagement.

The perspectives that underlie these studies deserve further comment. Current literature has evaluated policy-maker [7, 13, 16, 25, 32, 33] and researcher perspectives [12, 15, 17, 34–39]. However, the unique research environment of an academic researcher versus that of others warrants further attention, particularly those embedded within specialised graduate schools.

### The role of academia in EIDM

There are several examples of academia contributing to health policy development and reform, and innovative examples of engagement between institutes of higher education and decision-makers have emerged globally. These include the participation of researchers from academic institutions in technical government working groups [40], researcher engagement in multi-stakeholder consortiums or coalitions [41], student policy fellowship programmes [42], and the hosting of forums by academic institutions for deliberative policy dialogues [43, 44], amongst others. Additionally, personal and professional relationships between academics and decision-makers have been shown to affect EIDM through a knowledge brokering role [45–47].

Academia is considered to be an environment where critical thinking and theory utilisation are valued, where independent and credible research is honored, and where policy and practice-relevant inquiry is encouraged [12]. However, research in the academic environment is often accompanied by faculty responsibilities to teaching and service [10]. These multiple responsibilities and incentives that accompany them may at times be incongruent with respect to engaging with decision-makers, as engagement with decision-makers is often under-valued in appointments and promotions guidelines relative to peer-reviewed publications, grants, teaching and other core tasks [20, 48, 49].

The extent to which knowledge sharing occurs is likely to vary across contexts given the many channels through which academic researchers engage with decision-makers. For instance, a study on medical faculty interactions with non-academics in Canada demonstrated the importance of faculty profiles when considering personal interactions [50]. In the field of public health, where EIDM has the potential to impact millions of lives, it is important to understand how academic settings could contribute to enhancing researcher engagement with decision-makers in government. Schools of public health (SPHs) are especially critical to advancing KT given their applied orientation and mandate. Given that they can have a significant impact in influencing EIDM [23, 51, 52], factors affecting this potential need to be better understood [53].

### The context of SPHs in the United States of America

SPHs have played a foundational role in the emergence, differentiation and legacy of public health over the past century [3, 54]. In particular, SPHs have facilitated education and research in several areas of public health, including contagion, preventive health, health system design and delivery, primary healthcare and health promotion [55]. It is for these reasons, and the significant potential of public health, that Colgrove et al. characterise SPHs as “*essential infrastructure of a responsible society*” [31].

The importance of enhanced linkages between SPHs and government public health agencies in the United States was emphasised first in the 1980s by the Institute of Medicine Future of Public Health Report, which concluded that “*schools of public health have in recent years become somewhat isolated from the field of public health practice*” [56]. WHO also highlighted the necessary role of SPHs in transforming medical care systems [57]. SPHs responded with some results published in The Public Health Faculty/Agency Forum report [58] and the Pew Commission report [59].

Attention to the role of SPHs has not gone unnoticed as reflected in studies dedicated to studying their influence on decision-making [51, 60–63]. However, there is relatively limited understanding of the factors that influence engagement between SPH academic researchers and government decision-makers, how the individual and institutional factors interact, and how this may be different at the city, state, federal and global levels. This is critical for informing and building new interventions that can increase the bidirectional interchange between SPH faculty and decision-makers.

### Objectives and justification for this study

Utilising the extant research literature available, we sought to fill the abovementioned gaps so as to contribute to the discussion on interventions for supporting engagement of SPH faculty with decision-makers for purposes of knowledge exchange. Furthermore, we sought to explore relationships with decision-makers at four different government levels to reflect the local as well as global reach of the SPH in the study.

The purpose of this study was therefore threefold, namely, within the context of an SPH, to (1) explore the extent to which perceptions of individual, institutional, relational, research-related and funder-related factors affect academic faculty engagement with public health decision-makers, (2) involve faculty in identifying priorities to enhance engagement, and (3) utilise this insight to suggest recommendations for enhancement of facilitators and mitigation of barriers to engagement within the SPH.

## Methods

### Eligibility and recruitment

The Johns Hopkins Bloomberg School of Public Health (JHSPH) is located in Baltimore, MD, approximately 40 miles from Washington, DC. As the first and largest SPH globally, it hosts approximately 2340 students and 1500 faculty spanning 10 departments and 60 centres/institutes [64]. Between June and November 2016, all full-time faculty at JHSPH were invited to participate in the study. Engagement with decision-makers was not a requirement for participation nor was conducting research. After initial invitations were sent, those whose status was not verifiable, or were no longer employed by the institution, were reclassified as ineligible. This approach was taken to focus on existing full-time faculty members’ engagement behaviours. Three attempts at recruitment were made over the 6-month period, with invitations being sent out via email for the first two attempts and phone calls made for the final recruitment attempt. For those without valid or accessible phone numbers, a final attempt was made via email. Consent forms (for verbal consent if accepted) and a ‘frequently asked questions’ flyer on study goals and objectives was shared during follow-up invitations.

### Data collection

Data were collected through an interviewer-administered survey, which was conducted either via video Skype or in-person. The survey tool was adapted from a prior study [46] and refined by the study team. It was tested with faculty ineligible for the study but with similar characteristics to the target respondents. Survey responses were entered into Qualtrics Software [65]. All interviewers were members of the study team and performed simulations to ensure alignment in the approach and intent of the surveys. The survey took approximately 60 min, on average, to complete.

The survey consisted of three sections, as follows: (1) questions regarding respondents’ demographic and institutional affiliation, (2) perception of factors affecting their engagement with decision-makers, and (3) their suggested priorities for the SPH in enhancing researcher engagement with decision-makers. ‘Engagement’ was defined as interaction, communication, outreach or exchange that was active or underway during the period of study.

Respondents were also asked to provide the names of up to seven decision-makers at the Baltimore city, Maryland state, United States federal, and global levels with whom they are currently engaged (i.e. have some form of interaction or communication on a relevant issue in the 12 months preceding and up to the time of the study). Given that decision-making happens at various levels that may or may not be policy relevant, we focused the study on relationships with ‘decision-makers’. For the purposes of this study, a decision-maker was defined as someone who plays a key role in the administration (and leadership) of a government organisation that has the authority to influence or achieve specific health goals (e.g. administrative actions, guidelines and recommendations and policy decisions). These data were used to conduct a network analysis (reported elsewhere) [66] and generate the number of decision-makers each respondent was engaged with.

The list of facilitators and barriers was compiled from studies and reviews in the knowledge exchange and brokering literature that focused on academic researchers, advocates and decision-makers’ engagement experiences [13, 18, 23, 33, 67–69] as well as from responses received during instrument testing. In developing the survey, we noted that factors (facilitators as well as barriers) documented in the literature could be categorised within five loci of perceived control, namely individual characteristics, institutional environment, relational dynamics, research focus and funder policies, when considering knowledge exchange endeavours in contrast to knowledge utilisation. These categories were neither shared in advance with respondents nor were they presented in this order so as to minimise response bias. For each of the final list of 19 factors, respondents were requested to indicate, based on their personal experiences, whether it had been a facilitator, a barrier (i.e. whether its presence or absence was a hindrance), both a facilitator and a barrier, or neither.

The list of 11 priorities they could select from were based on recommendations included in prior studies of facilitators and barriers to engagement in academic settings, as well as proposed strategies at JHSPH [12, 13]. Respondents were asked to identify the top three priorities that the school should pursue at an institutional-level to promote faculty engagement with decision-makers; respondents were invited to suggest priorities that had not been listed.

### Data analysis

Respondents were anonymised and assigned a unique identification number prior to analysis. Some response variables related to faculty characteristics (e.g. areas of research expertise) were grouped into broader categories to assist with analysis. Categorical data with low response counts were collapsed for conceptual and analytic purposes (i.e. faculty position, number of years employed within the institution, race/ethnicity and department categories). Data were analysed using STATA 13.1 [70].

In order to understand whether influential factors varied by demographic attributes, research foci, network size (total number of government decision-makers they are connected to at the city, state, federal and global levels) or engagement experience, multiple χ^2^ tests or, where appropriate, Fisher’s exact test and univariate logistic regressions were performed. The characteristics included in analyses were faculty track and position, research area of expertise, the number of years employed within the institution and departmental affiliation. The relative risk of whether factor rating (i.e. the outcome variable) varied by network size was explored using logistic regression. To explore this association, a dichotomous variable was created for each factor affecting engagement; responses were coded as ‘1’ if they were identified as a facilitator and ‘0’ if they were identified as a barrier.

## Results

### Respondent characteristics

A total of 627 full-time faculty were eligible for inclusion in the study; this group consisted of tenure and non-tenure track faculty, as well as faculty in professorial and scientist career tracks. Within the context of this study, faculty in the professorial track have responsibilities primarily related to teaching and advising students, research and academic programme management, and include individuals with the titles Associate Professor, Assistant Professor and Professor. Faculty in the scientist track have responsibilities primarily related to research and project management and include individuals with the titles Research Associates, Associate Scientist, Assistant Scientist and Senior Scientist.

Of those, 211 (34%) participated in the study. Table 1 provides an overview of study respondents. Professors were the largest group amongst respondents (23%), followed by Associate Professors (12%), Assistant Professors (13%), Assistant Scientists (17%) and Research Associates (13%). Approximately 22% of respondents indicated holding joint appointments in two or more departments. With regard to career track, approximately 50% of respondents reported being in a professorial track and 46% in a scientist track. The distribution of faculty within the study sample was similar to the school-wide distribution reported in 2016 in terms of sex, race/ethnicity, position and career [71].

**Table 1:** Respondent characteristics

	Total Respondents (N)	Total Respondents (%)	Total Faculty in the SPH (%)
Respondents	211	34	
Sex			
Male	88	42	43
Female	123	58	57
Race/Ethnicity			
White	169	80	74
Black or African-American	7	3	5
Hispanic or Latino	4	2	4
Asian	20	9	16
Other	9	4	–
Faculty position			
Professor	49	23	25
Associate Professor	25	12	12
Assistant Professor	28	13	13
Senior Scientist	9	4	–
Associate Scientist	19	9	–
Assistant Scientist	35	17	–
Senior Research Associate	6	3	–
Research Associate	28	13	–
Other	12	6	–
Faculty track			
Professorial	105	50	49
Scientist	97	46	48
Number of years at the institution			
Less than 3 years	50	24	–
3 to 5 years	40	19	–
6 to 10 years	45	21	–
11 to 20 years	33	16	–
Greater than 20 years	43	20	–
Leadership position within the institution			
Yes	81	39	–
No	128	61	–
Current engagement with decision-makers in government			
Not currently engaged	57	27	–
Currently engaged	154	73	–
Baltimore City	44	21^a^	–
Maryland State	50	24^a^	–
United States Federal	95	45^a^	–
Global	77	37^a^	–
Other cities, states, counties	21	10^a^	–

^a^Indicates percentage of the total number of respondents (*n* = 211)

Respondents identified over 23 areas of research expertise; these subject areas were collapsed into 13 distinct categories. Several respondents identified more than one focus; the number of areas of research expertise per respondent ranged from 1 to 13. The top listed areas of expertise included infectious disease, epidemiology and disease control, maternal and child health, health behaviour, and non-communicable diseases.

Approximately 51% of respondents indicating having engaged with decision-makers prior to joining as faculty. Engagement levels increased at all levels of government after joining the university, with 83% of respondents reporting engaging with decision-makers since joining as faculty and 73% reporting being currently engaged.

### Perceptions of facilitators and barriers to engagement

Percent frequency data for respondent’s perceptions of facilitators and barriers to engagement with decision-makers are presented in Table 2. The complete phrasing of each factor included in the survey is provided in the column labelled, ‘Survey Phrasing’. A two-word contraction for each factor is provided in the column labelled, ‘Factor’ which is used henceforth.

**Table 2:** Distribution of responses for perceptions of facilitators and barriers to engagement

Factor	Survey phrasing	Facilitator n (%)	Barrier n (%)	Both n (%)	Neither n (%)
Individual characteristics					
Experiential knowledge	“Previous professional/practical experience in a decision-making environment”	115 (55)	20 (10)	6 (3)	70 (33)
Career stage	“Stage I am at in my professional career”	104 (49)	47 (22)	22 (10)	38 (18)
Faculty position	“My (academic or administrative) role/position at JHSPH”	114 (54)	27 (13)	23 (11)	47 (22)
KT skills	“Communication/knowledge translation/advocacy skills”	135 (64)	27 (13)	19 (9)	30 (14)
Institutional environment					
Departmental reimbursement	“Reimbursement by my department of costs incurred as a result of engagement”	41 (19)	33 (16)	2 (1)	135 (64)
Academic incentives	“Academic incentives (e.g. contribution to promotion and tenure) for engaging with decision-makers on research results/priorities etc.”	73 (35)	39 (19)	13 (6)	86 (41)
Workplace location	“Geographic location of my workplace”	99 (47)	31 (15)	18 (9)	63 (30)
Departmental culture	“Culture of pursuing policy- and/or practice- relevant research in my department”	116 (55)	29 (14)	11 (5)	55 (26)
Non-financial support	“Support (non-financial) from my supervisor/department”	122 (58)	27 (13)	13 (6)	49 (23)
Dedicated time	“Ability to carve out dedicated time for engagement with decision-makers”	65 (31)	90 (43)	20 (10)	36 (17)
Institutional affiliation	“Being affiliated with JHSPH (e.g. implied credibility)”	164 (78)	2 (1)	27 (13)	18 (9)
Relational dynamics					
Personal networks	“My own pre-existing relationships/networks”	139 (66)	17 (8)	10 (5)	45 (21)
Peer introductions	“Introduction to decision-makers by colleagues who have relevant relationships/networks”	164 (78)	7 (3)	8 (4)	32 (15)
Network culture	“Culture of policy engagement amongst my professional network outside the SPH”	126 (60)	12 (6)	9 (4)	64 (30)
Peer skills	“Support from colleagues with communication/knowledge translation/advocacy skills”	130 (62)	12 (6)	8 (4)	61 (29)
Research focus					
Research relevance	“Relevance of my research to pertinent policy issues”	164 (78)	5 (2)	23 (11)	19 (9)
Research implications	“Inclusion of contextual, economic or implementation-related implications of my research”	130 (62)	7 (3)	14 (7)	60 (28)
Funder policies					
External funding	“Financial support/compensation from external sources (funders) for engagement”	91 (43)	24 (11)	38 (18)	58 (28)
Funder requirements	“Requirements from funders regarding dissemination and policy engagement (e.g. lobbying restrictions, dissemination beyond publications, etc.)”	73 (35)	14 (7)	23 (11)	101 (48)

The primary facilitators identified by respondents ranged in responses from approximately 66% (Personal Networks; KT Skills) to 78% (Peer Introductions; Institutional Affiliation; Research Relevance). The range of responses for the top five barriers was much lower and much wider, from approximately 15% (Departmental Reimbursement; Workplace Location; Academic Incentives; Career Stage) to 43% (Dedicated Time).

#### Individual characteristics

*Experiential Knowledge* was acknowledged by 65% of faculty as a contributing factor to engagement. Respondents in more junior faculty appointments, specifically Research Associates, Assistant Scientists and Assistant Professors reported *Career Stage* and *Faculty Position* as a facilitator 20% and 18%, respectively, less frequently than the sample mean. Although 86% of respondents noted the importance of *KT Skills*, responses were similar across academic positions (i.e. there was no statistically significant association between academic position and whether this factor was identified as a facilitator or barrier, *p* = 0.701).

#### Institutional factors

Approximately 64% of respondents did not consider the availability or absence of *Departmental Reimbursement* to affect their engagement with decision-makers in government. There was, however, some variation in the role of this factor in by academic position, wherein Assistant Professors reported reimbursement from the department as a facilitator 20.1% more frequently than the sample mean.

Although the majority of faculty (52%) concurred that *Academic Incentives* affect engagement with decision-makers, 41% of respondents indicated that it was irrelevant. However, how academic incentives were rated varied by academic position. For instance, Assistant Professors reported the lack of academic incentives for engagement as a barrier 27.9% more frequently than the overall sample mean.

With Baltimore being approximately 40 miles from Washington DC, it is not surprising that approximately half (46.9%) of all respondents identified *Workplace Location* as a facilitator. Faculty in departments with research foci at the international level reported their location as a facilitator approximately 18.1% less frequently than the sample mean.

Respondents in departments whose work directly informed policy development and evaluation were found to report *Departmental Culture* as a facilitator approximately 16% more frequently than the sample mean, whereas respondents in departments that traditionally were not focused on policy reported it as a facilitator between 19% and 25% less frequently than the sample mean. Nevertheless, the extent to which *Departmental Culture* was considered a facilitator varied by department, and the association between whether it was reported a facilitator and respondents’ departmental affiliation was found to be significant (*p* = 0.027) (Table 3). There were also differences in terms of academic position, wherein Associate Professors reported *Departmental Culture* as a facilitator 20% less frequently than the sample mean. All Department Chairs who responded to the survey noted that it was an important facilitator. *KT Skills* and *Peer Skills* also varied between departments, with at least two departments reporting these as a facilitator 21% more frequently than the sample mean.

**Table 3:** Association between faculty characteristics and factor rating using Fischer’s exact test

	Factor	*P* value
Faculty characteristic (n)		
Faculty track (211)	Career stage	< 0.001
	Departmental reimbursement	< 0.001
	Faculty position	< 0.001
	Personal networks	0.020
Years at Johns Hopkins University (211)	Career stage	< 0.001
	Experiential knowledge	0.010
Primary department^a^ (143)	Peer introductions	0.022
	Departmental culture	0.027
	Funder requirements	0.033
Academic position^a^ (145)	Academic incentives	0.009
	Institutional affiliation	0.005
	Career stage	< 0.001
	Departmental reimbursement	0.003
	Faculty position	< 0.001
Areas of expertise^a^		
Infectious disease (81)	Peer skills	0.004
	Funder requirements	0.023
Non-communicable disease (43)	Knowledge translation skills	0.044
Maternal and child health (72)	Peer introductions	0.031
	Departmental culture	0.016
	Personal networks	0.005
Health services and systems research (63)	Research implications	0.002
	Knowledge translation skills	0.033
	Experiential knowledge	0.001
Environmental and occupational health (47)	Knowledge translation skills	0.017
Nutrition (25)	Peer skills	0.018
	External funding	0.009
	Knowledge translation skills	0.013
Health policy (63)	Workplace location	0.039
	Peer introductions	0.046
	Research implications	0.007
	Knowledge translation skills	< 0.001
	Personal networks	0.001
	Experiential knowledge	< 0.001
Health behaviour and promotion (64)	Academic incentives	0.041
	Departmental culture	0.027
	Knowledge translation skills	0.044
Biomedical sciences (38)	Departmental culture	0.007
	Funder requirements	0.030

This table only includes statistically significant findings with *p* < 0.050.

^a^For analytic purposes, only departments, academic positions and areas of expertise that accounted for greater than 10% of the sample were included in this analysis

The most commonly cited barrier, identified by 43% of respondents, was the availability of *Dedicated Time* for engagement. Informally, however, several respondents noted that it parallels how they feel about academic incentives, in that time is an important academic incentive and is therefore nested within other factors. Conversely, *Institutional Affiliation* was one of the three highest cited facilitators (78%) and did not have a statistically significant association with department (*p* = 0.749).

#### Relational dynamics

There were several factors that fell under the category of *Relational Dynamics*. Amongst these, each was noted as a facilitator as follows: *Personal Networks* (66%), *Peer Introductions* (78%), *Network Culture* (60%) and *Peer Skills* (62%). However, between 15% and 30% did not consider these as relevant factors at all. The extent to which respondents identified *Personal Networks* and *Peer Introductions* as facilitators varied by department.

#### Funder policies

Less than half of respondents (43.1%) identified *External Funding* as a facilitator for engagement. *Funder Requirements* could have been considered a facilitator in the event that funders include engagement as a condition of funding, require dissemination of results and/or provide resources for engagement. It could have also been perceived as a barrier in instances where funders specifically prohibit engagement or do not provide resources to offset the costs of dissemination. *Funder Requirements* was found to have a statistically significant association with respondents’ departmental affiliation (*p* = 0.033).

### Faculty characteristics predictive of perceptions of facilitators and barriers to engagement

Research area of expertise, faculty track, faculty rank, engagement with decision-makers and number of relationships with decision-makers were faculty characteristics that were significantly associated with factors identified as facilitators.

#### Faculty position and track

The relevance of a factor also varied by faculty track. For example, there were statistically significant differences in whether *Career Stage*, *Faculty Position* and *Personal Networks* were identified as facilitators or barriers between faculty in scientist and professorial tracks (Table 3).

#### Research areas of expertise

The relevance of a factor often varied by respondents’ areas of research expertise as seen in Table 3. For example, there were statistically significant (*p* < 0.05) differences in whether *Personal Networks* were identified as a facilitator or barrier between respondents that selected health policy and maternal and child health as their research areas of expertise.

#### Engagement with decision-makers

For most factors, there were statistically significant (*p* < 0.05) differences between faculty that were currently engaged with decision-makers relative to those that were not (i.e. *Career Stage*, *Institutional Affiliation*, *Peer Introductions*, *Peer Skills*, *External Funding*, *Workplace Location*, *Funder Requirements*, *Faculty Position*, *KT Skills*, *Personal Networks*, *Network Culture*, *Experience Knowledge*, *Research Relevance*, and *Research Implications*).

#### Decision-maker network size

As a faculty member’s network size increased, they were more likely to identify the following factors as facilitators rather than barriers: *Career Stage*, *Institutional Affiliation*, *KT Skills*, *Personal Networks*, *Network Culture* and *Experiential Knowledge* (Table 4).

**Table 4:** Relative risk of factor rating by number of alters identified

Factor	Relative risk	95% Confidence interval
Career stage	1.06	1.01–1.11
Institutional affiliation	1.19	1.09–1.28
Knowledge translation skills	1.07	1.01–1.12
Personal networks	1.09	1.03–1.14
Network culture	1.07	1.02–1.12
Experiential knowledge	1.14	1.06–1.21

This table only includes statistically-significant findings with a 95% CI that does not include 1

#### Priorities

A complete list of priorities to enhance engagement with decision-makers, their contracted code and their percent frequency is provided in Table 5. While priorities varied slightly across departments, the top three priorities for the SPH as indicated by respondents were *More Incentives*, *Supplemental Funding* and *Enhancing Culture*.

**Table 5:** Percent frequency of institutional priorities to enhance engagements with decision-makers

Priority	Code	n (%)
Create more academic incentives for such engagement (e.g. contribution to promotion and tenure)	More incentives	105 (49.8)
Provide supplemental funding for knowledge translation and/or facilitate financial support/compensation from external sources	Supplemental funding	88 (41.7)
Enhance/inculcate a culture of pursuing policy- and/or practice-relevant research	Enhancing culture	83 (39.3)
Strengthen faculty (and student) capacity in communication/knowledge translation/advocacy skills	Strengthening knowledge translation skills	68 (32.2)
Assist faculty with building and maintaining important relationships/networks	Networking assistance	63 (29.9)
Mediate/broker introductions to decision-makers by colleagues who have relevant relationships	Mediating relationships	61 (28.9)
Facilitate faculty in carving out dedicated time for such engagements	Allocating time	54 (25.6)
Promote and support experience in a decision-making environment	Value experience	29 (13.7)
Hire more faculty and staff with training in communication/knowledge translation/advocacy skills	Hire knowledge translation faculty	24 (11.4)
Leverage technology to engage remotely with decision-makers	Leverage technology	16 (7.6)
Provide more mentoring/moral support from supervisors	Mentoring support	16 (7.6)

While the priorities varied across the departments in terms of where they ranked, there were many similarities. For instance, *More Incentives* ranked in the top three for all ten departments and ranked first for half of all departments. *Supplemental Funding* scored in the top three for seven of out of ten departments. Of note, *Mentoring Support* and *Leverage Technology* consistently scored in the bottom three across departments. While *Strengthening KT Skills* was noted by 32% of all faculty, 80% of department Chairs reported it as a priority. Similarly, *Enhancing Culture* was listed by 39% of faculty overall and 80% of department Chairs. Although responses differed by various respondent characteristics, logistic regression tests found that primary departmental affiliation, academic position, years within the institution and number of alters were generally not predictive of priority selection.

## Discussion

This study demonstrates that individual characteristics, institutional factors, relational dynamics, research foci, as well as funder policies affect academic researchers’ engagement with public health decision-makers. Moreover, these data suggest that factors differ in their status as a facilitator or barrier (in terms of respondents’ institutional position, rank and department), as well as individual experiences (areas of research expertise, engagement with decision-makers and network size) similar to some of the findings from Ouimet et al. [50].

The most commonly cited facilitators identified in this study spanned relational, individual, institutional and research-related characteristics, whereas the most commonly cited barriers focused on institutional and individual factors. These findings are consistent with Oliver et al.’s [13] review of facilitators and barriers to knowledge exchange. The circumstances of academic researchers in a university setting are different to those of the researchers within other contexts. We have therefore sought to see if the barriers and facilitators resonate within this group and whether we can reinforce existing studies, and if not, to provide an alternate perspective. This study differs in that, instead of viewing individual as well as institutional determinants as separate, we explore them in conjunction to see if and how individual characteristics are associated with responses to institutional factors.

### Institutional characteristics

Faculty responses to the various factors affecting engagement often differed by department, implying that there is variation in how departments either explicitly or implicitly encourage, support and facilitate their faculty to engage with decision-makers and/or how faculty in distinct departments perceive engagement. These findings are supported by earlier research on the mediating role of incentives and professional and time costs in knowledge exchange processes [72]. Although departmental affiliation may play an important role in faculty engagement, our findings suggest faculty’s research area of expertise was also predictive of how factors were rated. Accordingly, discipline and/or domain-specific norms may also affect faculty perceptions of facilitators and barriers to engagement. For example, faculty with interests in health policy, services and systems research were found to be more likely to rate *Personal Networks* and *Experiential Knowledge* as facilitators, which may reflect the unique relational or political norms that exist within the health policy domain.

The variation in responses regarding faculty institutional affiliation suggests that, while an academic institution’s brand and reputation can be generally positively viewed, there may be negative experiences or intentions associated with academia that affect the credibility or opportunity for faculty influence. These negative experiences may be attributable to stakeholder experiences regarding ‘parachute’ consultants, prioritisation of foreign researchers at the expense of local expertise, or past activities in local or international communities that have undermined trust.

### Individual characteristics

Differences in responses and experiences between faculty in professorial and scientist tracks, as well as within each track, suggest the professional norms governing career trajectories may differ. Respondents placed a high value on *Personal Networks*, which suggests that the importance of relationships with decision-makers has been recognised as critical to influencing decision-making. However, the fact that most senior staff noted the value of their own networks whereas junior staff valued the introductions to relevant colleagues indicates that particular attention needs to be paid to assist faculty in more junior positions to build, nurture and maintain their networks. *Peer Introductions* being one of the top ranked facilitators further supports this assertion. Establishing partnerships between researchers and decision-makers, as well as increasing contact and communication between them have been found to drive engagement [13, 21, 27] and enhance the ‘embeddedness’ of an organisation [73]. Enhancing relationships with key decision-makers is an area that SPHs – and academic institutions in general – should therefore encourage, support and broker.

Faculty that were engaged with decision-makers were more likely to consider a broad range of institutional and individual factors as facilitators than faculty peers who were not engaged. This finding suggests that faculty perceptions of engagement may depend on exposure to successful engagement. Not surprisingly, faculty that had a larger number of relationships were more likely to identify *Personal Networks* as a facilitator.

Faculty responses regarding *Funder Requirements* suggest there is variation in whether funders support or encourage engagement with decision-makers and that variation in these policies is apparent in terms of domain. Greater support, guidance or requirements from funders may therefore enhance researcher engagement.

### Priorities

The top priorities identified by respondents emphasised creating additional incentives for engagement through changes to promotion and tenure criteria, supplemental funding for engagement, and reforms to departmental and school culture regarding engagement. These findings were consistent with *Academic Incentives* and *Departmental Reimbursement* being identified as barriers by 18.5% and 15.6% of respondents, as well as other studies where appropriate incentives as well as funding appeared amongst the most commonly cited barriers [20, 22, 74].

We note that the identified priorities did not always align to the reported facilitators/barriers as expected. For instance, although *Time* was the most frequently cited barrier, only 26% prioritised *Allocating Time*. This may be a reflection of the institutional focus of the priority response options compared to the individual focus of the facilitator and barrier factors. The fact that priorities were consistent across a variety of faculty characteristics (e.g. position, number of years within the institution and departmental affiliation) suggests future interventions should target both specific groups (e.g. faculty in scientist tracks, junior faculty, faculty in certain domains) and the school as a whole. Innovative technology and staffing-related priorities were not well ranked, which suggests such strategies may be underappreciated. These findings were consistent with Brownson et al.’s [60] identification of seven strategies that SPHs may implement to enhance the impact of public health research.

## Implications

The fact that several facilitators as well as barriers to engagement that are relevant to an SPH have been highlighted through this study begs dedicated attention by the institution of the study focus as well as SPHs more widely. Although the reasons why faculty in this study considered certain factors facilitators or barriers as well as their explanations for why the chosen priorities emerged in the way that they did are not known, future research that addresses this question would be valuable.

Interventions affecting faculty engagement with decision-makers require a systemic approach that considers approaches that span at least four of the five categories of influences articulated in the framework (e.g. individual, institutional, relational, research related). Of note, any interventions aimed at enhancing incentives and/or faculty skill development need to be designed with a view that these would be integral to the broader goal of organisational effectiveness [75], relevance, embeddedness and connectivity. Furthermore, given the variations in results by department, research area of expertise, academic position, career track and rank, any interventions should be tailored to these influences. SPHs would benefit from testing of such interventions empirically to ascertain their utility and effectiveness in enhancing engagement between faculty and decision-makers – government or otherwise.

Training and mentorship approaches targeting both faculty and doctoral or masters-level public health students may also help mitigate barriers related to relational skills and individual characteristics [76]. Some lessons could be learned from the University of Cambridge Policy Fellows Programme [42] as well as the University of North Carolina’s Thorp Engaged Scholars Program [77].

Like many other SPHs [51, 61, 77], JHSPH has several initiatives underway that reflect its appreciation of EIDM and the role that the institution could play in advancing EIDM. These include advocacy skill-building workshops, seminars to develop and build consensus around policy recommendations, small funding opportunities for the development of dissemination products, placements for decision-makers-in-residence, and creation of a ‘Professors of Practice’ title for faculty with extensive policy-making experience. However, given that they do not necessarily align with faculty-identified priorities indicates that perhaps well-intentioned interventions could benefit from further faculty input. Stakeholder engagement greatly increases acceptability and uptake of institutional change and interventions [78, 79]. The results of this study may assist with providing the first step in engaging stakeholders (faculty) in contributing to institutional priority-setting. Particular attention to strengthen and sustain facilitators as well as alleviate barriers at the institutional as well as individual levels should be a complementary priority henceforth.

Our study suggests that, if indeed SPHs and academic settings intend to further affect change in policy, practice or public goals through the research and expertise embedded within their institutions, they should consider (1) periodically measuring engagement with decision-makers within their institutions in order to understand what the facilitators and barriers are and how they evolve; (2) enhancing interventions to build individual capacity in KT and research communication, taking faculty characteristics into account; (3) institutionalising a culture of EIDM that prioritises academic incentives and related practices for engagement both school-wide and within specific departments, research domains and career tracks; and (4) creating a strategy to expand and nurture trusted, relevant networks and relationships with decision-makers.

### Strengths and limitations

As is the case with all research, there are strengths and limitations of the study. One of the most notable strengths is the application of existing literature on individual, institutional, and relational facilitators and barriers to decision-maker engagement to a specific academic environment (i.e. a SPH). Understanding whether current examination of facilitators and barriers in KT in the literature are relevant to the context of a SPH was an important first step. Furthermore, we have been able to delineate a novel five-point categorisation of the facilitators and barriers as they are relevant to knowledge exchange in contrast to knowledge utilisation, providing a different focus within the KT cycle.

Contrary to recommendations for involving stakeholders in priority-setting exercises [78, 79], there are no studies that we are aware of that have used academic faculty to contribute to priority-setting in a SPH. By inviting respondents to contribute to SPH priority-setting, we believe that the list that emerged is more reflective of what should be considered by leadership at JHSPH, and perhaps at other SPHs as well. In addition, by stratifying facilitators and barriers by academic and administrative position, areas of research expertise, network connectedness and other factors, this study clarifies important differences in terms of individual and contextual factors.

The incentives, time considerations and commitments for full-time faculty are likely different than those for part-time faculty. By limiting the study to full-time faculty only, we may be underestimating the impact of the various factors identified on faculty ability to engage with decision-makers. In addition, part-time faculty may report distinct facilitators and barriers given the many unique responsibilities and time constraints that characterise part-time faculty roles. If future studies include both cadres of faculty, it would be interesting to note differences, if any, of how the various factors affect their engagement with decision-makers.

While there was a deliberate limit in scope – due to feasibility – to faculty relationships with government decision-makers as a proxy for influence, we recognise that other actors influential in health policy (non-governmental organisations, philanthropic organisations, civil society organisations and advocacy coalitions) have not been captured.

As oftentimes is the case with surveys, aspects such as ‘engagement’ or ‘decision-maker’ may have been subject to interpretation even though definitions were provided in advance. To minimise misinterpretations we (1) provided a list of FAQs with the invitations to clarify the purpose, inclusion criteria and format of the study and (2) administered the surveys personally rather than electronically in order to respond to questions arising from respondents. Given that this was a researcher-administered survey, it is possible that there was also some level of social desirability bias. For instance, there may have been cases where more distant or past relationships were mentioned in order to provide an impression of a larger network. In addition, faculty perceptions of facilitators or barriers to engagement may differ from actual structural facilitators and barriers within the institution.

Given that 22% of our respondents indicated holding positions in more than one department (or school) leads us to interpret our results with caution given that faculty working across multiple departments are likely to be influenced diffusely rather than explicitly by the cultures of those departments.

Response rates differed by departmental affiliation and were substantially lower amongst departments focused on basic science and biostatistics. Accordingly, the sample size for such departments are small and the regression results have wide confidence intervals. In addition, given the response rate of 34%, these findings may not be fully representative of the overall faculty population. For example, it is possible that faculty that were highly engaged or not engaged self-selected out of study on account of the perception that their work was outside the scope of the study and/or that their contacts were too sensitive.

As is the case with surveys, this study captures experiences at one point in time. In order to capture more longitudinal change in the institution as well as changes in perceptions if indeed some of these interventions are considered, surveys such as these would need to be repeated periodically.

Lastly, this study focused on a single SPH in order to capture the full breadth of experiences of academics engaging with the decision-making process within one context and the facilitators and barriers that mediate those experiences. Accordingly, study findings may not be generalisable to SPHs or other graduate institutions with fewer faculty or less research funding. Similarly, generalisability may be affected by the geographic location of Johns Hopkins, which is in close proximity to federal government offices in Washington, DC.

## Conclusion

SPHs are increasingly being recognised as important contributors of human, social and intellectual capital relevant to public policy decision-making. While there may be initiatives underway to enhance appreciation of this role as well as strengthen capacity for engagement, this study demonstrates that individual characteristics, institutional factors, relational dynamics, research foci and funder policies interact in a complex manner with respect to academic researchers’ engagement with public health decision-makers. Moreover, these data from one SPH suggest that factors differ in their status as a facilitator or barrier in terms of institutional characteristics in conjunction with, and not separate from, individual experiences. Particular attention to strengthen and sustain facilitators as well as alleviate barriers at the institutional as well as individual levels should be a priority. Assuming that funder policies with respect to dissemination and decision-maker engagement requirements are out of the realm of institutional control, it would benefit SPHs to consider an integrated approach to build on their strengths as well as diminish their challenges in the other four areas. Of note, any interventions aimed at faculty skill development need to be designed with a view that these would be integral to the broader goal of organisational effectiveness.

## Abbreviations

EIDM: Evidence-informed decision-making
JHSPH: Johns Hopkins Bloomberg School of Public Health
KT: Knowledge translation
SPH: School of public health
